# The impact of a novel digital sun protection campaign on sun‐related attitudes and behaviours of healthcare workers: A prospective observational study

**DOI:** 10.1002/ski2.256

**Published:** 2023-06-08

**Authors:** Emma Porter, Siobhan Rafferty, Michelle Dolan, David McMahon, Ali Sheikhi, Sinead Field, Evelyn Power

**Affiliations:** ^1^ Department of Dermatology University Hospital Limerick Limerick Ireland; ^2^ Irish Skin Foundation Dublin Ireland; ^3^ Health Research Institute University of Limerick Limerick Ireland

## Abstract

**Background:**

13 000 cases of skin cancer are diagnosed annually in Ireland, with ultraviolet (UV) radiation exposure the strongest risk factor. Public health primary prevention campaigns focus on encouraging safe sun protective measures and skin cancer awareness. We designed a novel, digitally‐animated hospital‐based campaign targeting all aspects of sun‐protective behaviour.

**Objectives:**

To explore the sun‐protective attitudes and behaviours of healthcare workers, and the effect of a digital hospital‐based campaign on these findings.

**Methods:**

This was a cross‐sectional prospective observational study involving hospital staff across the six hospitals that form the University of Limerick Hospital Group (ULHG). A two‐phase online survey, the first taking place before campaign launch, and the second upon campaign completion. The digital campaign was displayed across all hospital sites from June to September 2021. Surveys comprised questions on sun‐related attitudes and behaviours, including the internationally validated Sun Exposure Protection Index (SEPI) questionnaire.

**Results:**

Eight hundred fifty‐seven staff members completed survey 1 and 704 completed survey 2; 90% in each were female; 79% were aged 25–54; 71% reported skin types I–III. Best sun‐protective habits pre‐campaign included sunscreen use and avoiding sunburn, while wearing hats, protective clothing and seeking shade were least adopted. For 177 matched participants, there were small improvements in SEPI scores. SEPI Part 1 scores, reflecting improved risk behaviour, pre‐campaign had a median of 11 (IQR 7), and 11 (IQR 6) post campaign. SEPI Part 2 scores, reflecting readiness to adopt sun‐protective behaviours, improved from median 7 (IQR 8) to 6 (IQR 6). Post‐campaign, small improvements were seen across some individual sun‐protective behaviours and attitudes, particularly reducing time spent in the midday sun and on sun‐seeking holidays, and improved readiness to seek shade and reduce sunbathing; 79% of all respondents post‐campaign (*n* = 556) reported raised skin cancer awareness, and 65% (*n* = 458) said it influenced them to discuss sun protection with others.

**Conclusions:**

Positive improvements in attitudes and behaviours related to sun protection were seen following the digital campaign in this population of healthcare workers. These improvements along with increased willingness to discuss sun protection with others, including patients, has the potential to further benefit wider society, and supports future digital health promotion initiatives.

## Graphical abstract

This study examined the effect of a novel digital health promotion campaign surrounding sun protection on sun‐related attitudes and behaviours in a population of Irish healthcare workers. Following the campaign, many of these improved, most significantly with regard to sunbathing behaviours and seeking shade. Confidence in communicating sun protection messaging increased. The findings of this study provide a unique insight into awareness of this population of safe sun behaviour, and demonstrates the effectiveness of a targeted digital campaign on these.

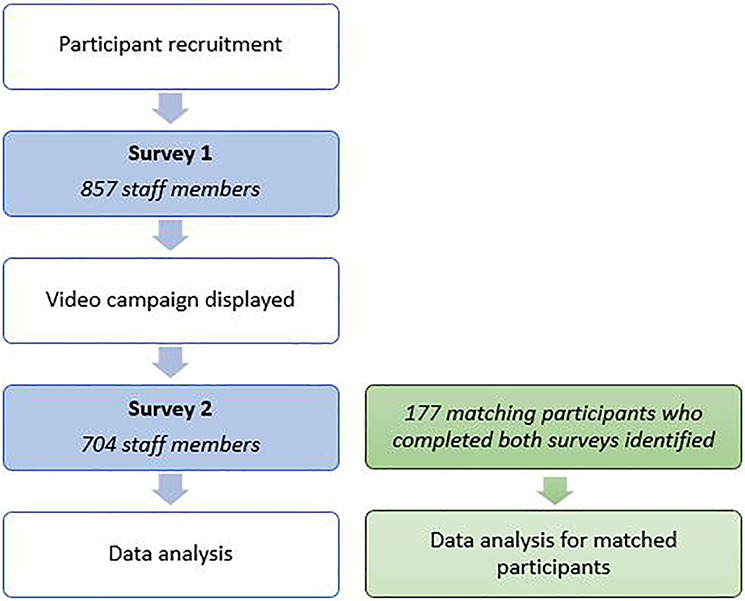

**What is already known?**

- Ultraviolet radiation is a risk factor in skin cancer development, and primary prevention campaigns focus on encouraging safe sun protective measures and skin cancer awareness
- Healthcare workers represent a unique cohort of the population as health‐related messaging can have an impact both on the individual and the patients they encounter

**What does this study add?**

- This study provides a detailed insight into the sun‐related attitudes and behaviours of healthcare workers in Ireland
- Amongst this population, sun‐protective practices were mixed, with high uptake of sunscreen use however less favourable adoption of seeking shade, protective clothing and hats
- Our campaign, delivered over one summer season, had a positive impact on self‐reported sun protective habits, skin cancer awareness and confidence in discussing sun protection with others

## 1 INTRODUCTION

Ireland has one of the highest incidence rates of melanoma globally, ranking 10th in the Global Cancer Observatory in 2012.^1^ Rising incidence of melanoma has been observed worldwide particularly among populations with a large cohort of at‐risk fair and light‐skinned individuals.^2^ Non‐melanoma skin cancers similarly have increased in incidence, including in younger age groups.^3^ Ultraviolet (UV) radiation exposure is the most important risk factor.^4^ Several studies have been conducted in relation to UV protection behaviours, attitudes towards tanning and skin cancer risk.^5^, ^6^, ^7^, ^8^ Knowledge of skin cancer is positively associated with sun protection behaviours.^6^

Public health efforts are directed towards encouraging those at risk to adopt sun‐safe practices. Digital interventions in promotion of safe sun‐protective behaviours have become increasingly utilised and have shown encouraging results including a positive impact on skin cancer outcomes.^7^ The SunSmart campaign, launched in Australia in 1988, has proved successful in preventing skin cancer^8^ and become a widely utilised and internationally recognised skin cancer prevention campaign. Ireland's National Skin Cancer Prevention Plan^9^ incorporates the SunSmart messaging of Slip, Slop, Slap, Slide, and Seek Shade.

Healthcare workers represent a unique cohort in health promotion, as this group has direct involvement in patient care and may contribute to improving prevention and early diagnosis of skin cancer. Educating healthcare staff about safe sun behaviours and improving attitudes surrounding sun tanning creates not only an opportunity to promote this messaging in this group but to further encourage sharing advice with patients.^10^, ^11^, ^12^, ^20^, ^21^ This study explores the attitudes and behaviours relating to sun protection and tanning, and awareness of skin cancer in Irish healthcare workers.

## 2 MATERIALS AND METHODS

**FIGURE 1:**
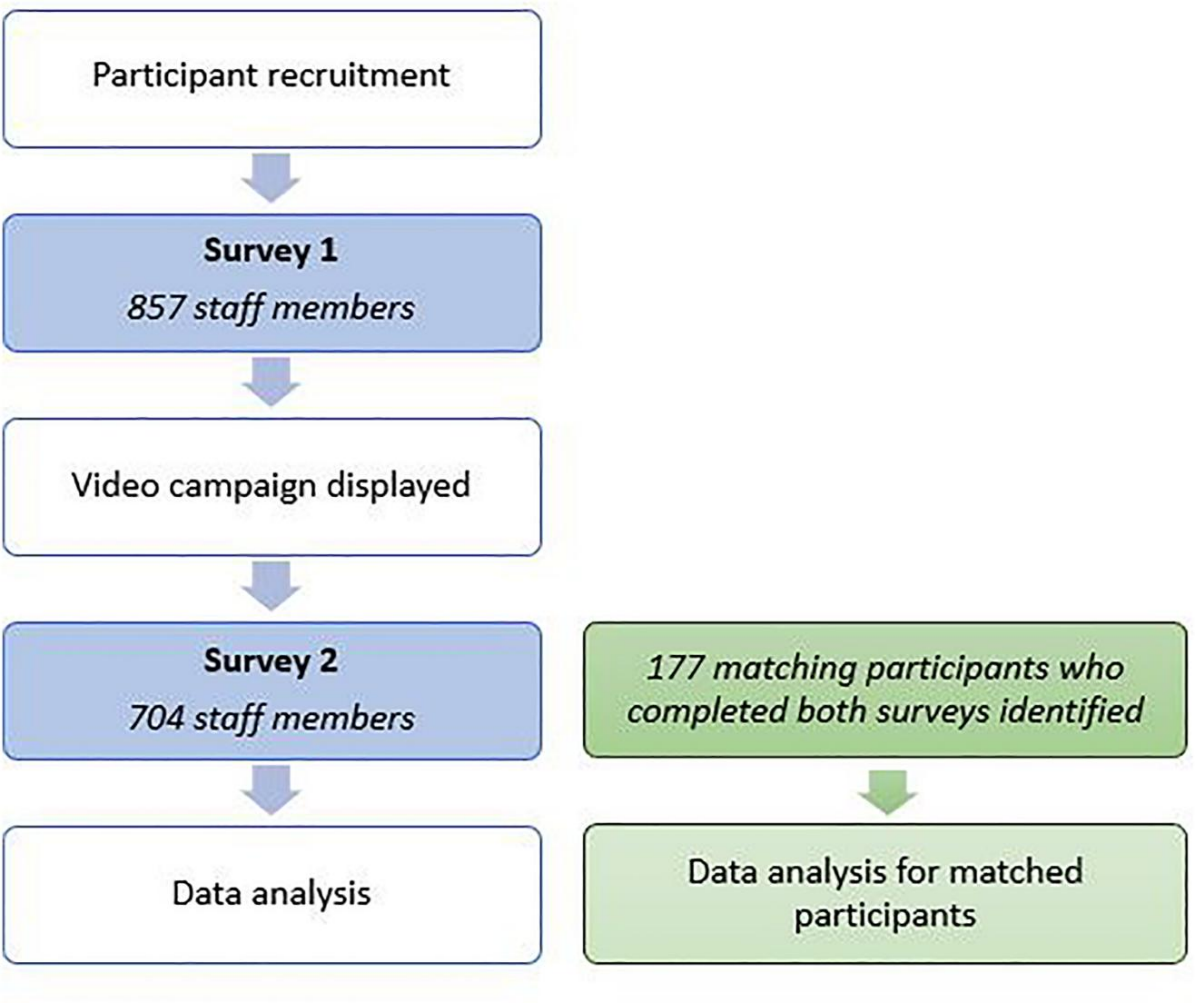
Flowchart summary of study methodology.

This study was open to all staff members of the University of Limerick Hospital Group (ULHG), comprising six hospitals located in the mid‐West of Ireland with over 4500 employees. The study was divided into two phases, the first survey taking place in early summer 2021, prior to introduction of a video campaign for sun protection and safety awareness. At the end of the video campaign, in September 2021, the survey was repeated (Figure 1).

### 2.1 Recruitment of participants

Participants were recruited to the first survey through staff email, staff mobile applications, posters, hospital social media and word of mouth. On‐site posters were displayed in areas of high staff foot traffic such as canteens, staff rooms and rest areas. Participation was incentivised with advertisement of a competition to win a selection of prizes for participation in the study. The survey was advertised and open for a period of 6 weeks. Thereafter, the digital campaign was launched. Towards the end of the campaign, the second survey was advertised to staff and invitations were sent to those who had completed part one. This survey remained open for 6 weeks.

### 2.2 Video campaign

**FIGURE 2:**
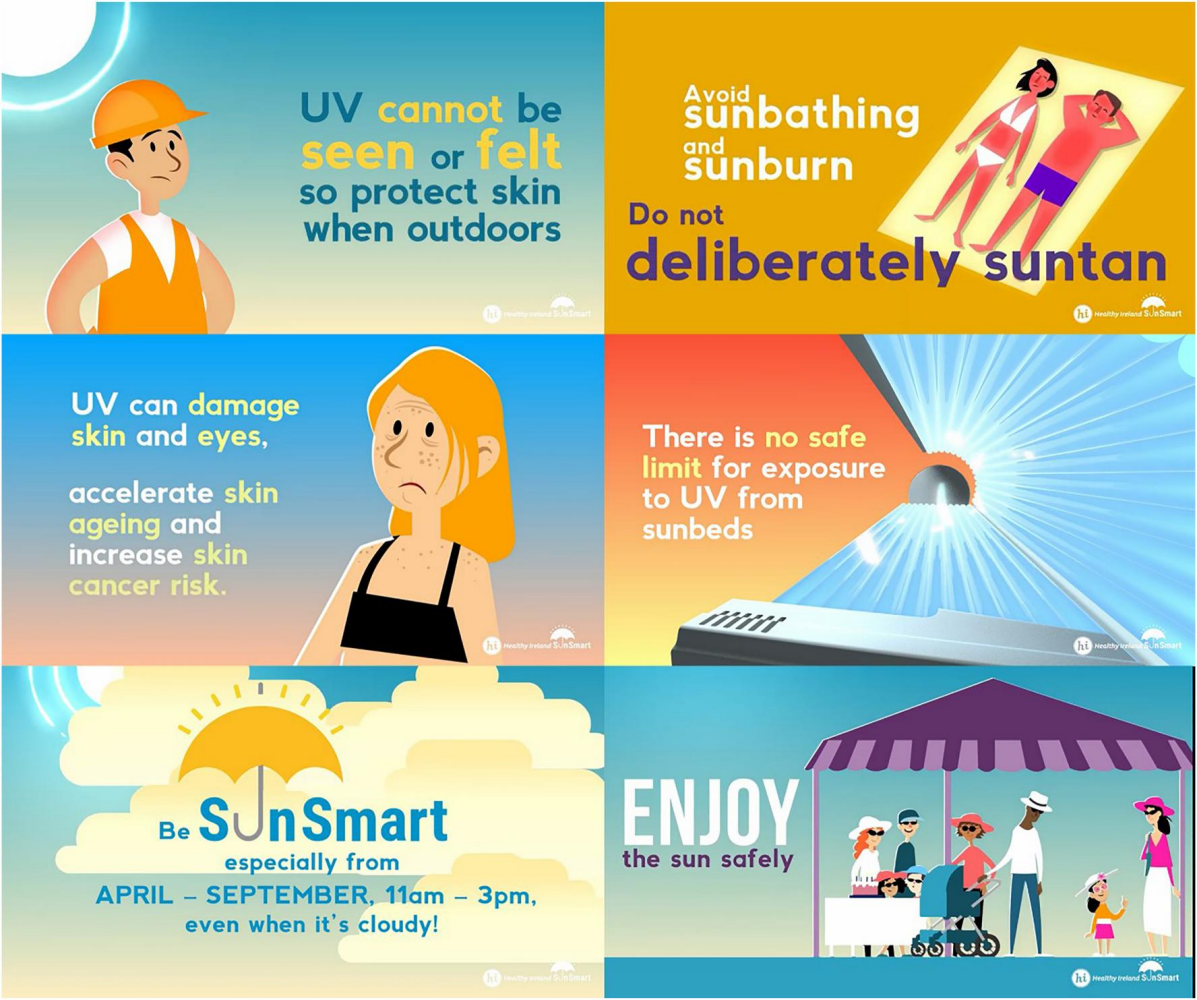
Images of elements of the animated video campaign created in collaboration with the Irish Skin Foundation. Themes included children's exposure to UV, outdoor work, sun tanning, sun bed use, outdoor leisure, and general sun protection. Each video emphasised the 5 S's of the SunSmart message—Slip, Slap, Slop, Slide and Seek Shade.

Working closely with the Irish Skin Foundation, we created a series of five informational animated videos (Figure 2). These focused in particular on targeting the four high‐risk groups set out by the National Skin Cancer Prevention Plan 2019–2029: children and young people, outdoor workers, those involved in outdoor leisure activities and sunbed users. All videos included general sun protection advice incorporating the multicomponent SunSmart messaging of Slip, Slop, Slap, Slide, and Seek Shade – especially from April‐September between the hours of 11 AM–3 PM, even in cloudy weather. Each video was of short duration (60–90 s). Videos were displayed across visual display units and communication screens across all hospital sites, located in areas of high staff use and public spaces in the hospital. Links to the videos were also included in staff newsletter emails, on staff mobile applications and on social media webpages of ULHG, the Irish Skin Foundation, and NCCP. The video campaign and its messaging were further advertised by clinicians for health promotion amongst the general population through media segments on local and national radio.

### 2.3 Survey design

Detailed cross‐sectional surveys were conducted online using SmartSurvey™ software. Questions were designed to capture sun habits and sun protection behaviour. Demographic data collected included age range, sex, educational level, marital status, Fitzpatrick skin type, and nature of work in the hospital for example, nursing, medical, administrative. In the second survey, conducted at the end of the summer campaign, additional items on the questionnaire included those pertaining to the reception of the campaign, whether it had been seen and where, and whether participants considered it to have affected their attitudes and behaviours surrounding sun protection. Questions surrounding sunbed use were added in the second survey. A full copy of the questionnaires used are available as Supplementary files.

### 2.4 Sun exposure and protection index (SEPI)

Questionnaires incorporated the Sun Exposure Protection Index (SEPI), which is a validated questionnaire for the assessment of solar ultraviolet radiation (UVR) exposure and sun protection behaviour.^13^ It has been validated in multiple languages and across numerous countries and populations worldwide, with varying UV radiation exposure and population demographics.^14^, ^15^, ^16^ It incorporates two parts: the first part (SEPI‐1) assesses sun habits and sun protection behaviour. The first part consists of eight questions with five‐point Likert scale responses (corresponding to scores of 0–4 points); a higher score corresponds with higher risk behaviour. The second part (SEPI‐2) evaluates readiness to increase or improve safe sun behaviour. This comprises five questions with responses based on the stages of change according to the transtheoretical model of behaviour change (maintenance, action, preparation, contemplation and precontemplation). A higher SEPI‐2 score indicates lower propensity to improve sun protection.

### 2.5 Cyber‐attack

In May 2021, the Irish health service was significantly impacted by the country‐wide cyber‐attack on the Health Service Executive information technology systems. As a result, access to hospital computers was heavily restricted, with complete loss of access to Internet, email servers and patient management and laboratory systems. This caused a delay of more than 3 weeks to the commencement of our first survey and campaign launch, which had been planned for early May 2021.The participant recruitment process was restricted to physical posters in hospital sites and word of mouth – further limited by ongoing COVID‐19 measures – which impacted on our ability to guarantee the same participants completed both phases of the study.

### 2.6 Statistical analysis

Data collected from SmartSurvey™ were analysed using IBM SPSS Statistics software (version 28.0.0). Survey participants who completed both surveys were identified as a subgroup (*n* = 177) by an external statistician using a coding system based on email addresses entered at the end of each survey by participants, to which the authors were blinded. As data were not normally distributed, median scores and interquartile ranges are displayed, with non‐parametric Wilcoxon‐Signed Ranks testing performed to assess change in all variables of the SEPI questionnaire.

## 3 RESULTS

Of 4500 staff invited to participate, 857 completed survey 1 and 704 completed survey 2; 90% of respondents in each were female. The most predominant staff groups were nursing (40% and 41.4% in surveys 1 and 2 respectively, and 48.6% in the subset who completed both surveys) and administration (28.4% and 29% in surveys 1 and 2 respectively, and 20.3% in the subset who completed both surveys). The majority were aged 25‐54 and reported skin types I‐III, with a similar distribution across surveys 1 and 2, including in those who completed both. Participant demographics are outlined in Table 1.

**TABLE 1:** Demographics of participants for each survey.

		Survey 1 (*n* = 857) *n* (%)	Survey 2 (*n* = 704) *n* (%)	Subset of participants who completed both surveys (*n* = 177)
Sex	Female	771 (90)	634 (90.1)	157 (88.7)
Age	18–24	48 (5.6)	30 (4.2)	8 (4.5)
	25–34	208 (24.3)	172 (24.4)	51 (28.8)
	35–44	226 (26.4)	204 (29.1)	46 (26)
	44–54	248 (28.9)	199 (28.4)	51 (28.8)
	55+	127 (14.9)	99 (14.1)	21 (11.9)
Skin type	I	167 (19.5)	125 (17.9)	40 (22.6)
	II	232 (27.1)	215 (30.6)	43 (24.2)
	III	216 (25.2)	149 (21.2)	46 (26)
	IV	143 (16.7)	129 (18.4)	30 (16.9)
	V	80 (9.4)	69 (9.9)	15 (8.5)
	VI	19 (2.2)	17 (2.4)	3 (1.7)
Occupation	Nursing	339 (40)	290 (41.4)	86 (48.6)
	Medical	37 (4.3)	32 (4.6)	12 (6.8)
	Allied health staff^a^	148 (17.3)	121 (17.3)	30 (16.9)
	Administration	243 (28.4)	204 (29)	36 (20.3)
	Catering	19 (2.2)	15 (2.1)	4 (2.3)
	Maintenance	5 (0.6)	6 (0.9)	2 (1.1)
	Other	66 (7.7)	36 (5.1)	7 (4)

^a^ Allied health staff included pharmacists, physiotherapists, laboratory staff, occupational therapists, paramedics/ambulance staff, social workers and care assistants.

### 3.1 Sun exposure and protection index (SEPI) scores

One hundred seventy‐seven matched survey participants were identified across both surveys. Tables 2 and 3 illustrate and compare median scores for SEPI questionnaire items in Surveys 1 and 2 for this group for SEPI Part 1 and SEPI Part 2 respectively. Statistically significant improvement was observed in the total score for SEPI Part 2 – reflecting improved readiness to adopt safe sun behaviours. While most domains had at most minor improvements in difference between median scores, particular items of positive change included reduced duration spent in the midday sun, reduced holiday time with the intention of spending time in the sun, improved readiness to seek shade and reduce sunbathing.

**TABLE 2:** Results from the Sun Exposure and Protection Index (SEPI) part 1 questionnaire items for surveys 1 and 2 for matched participants (*n* = 177).

Question/statement	Response categories	Frequency in survey 1, *n* (%)	Frequency in survey 2, *n* (%)	Survey 1 median score (IQR)	Survey 2 median score (IQR)	Change in scores, median (IQR)	*p* value
How many times have you been sunburnt during the last 12 months?	None	82 (46.3)	71 (40.1)	1 (1)	1 (1)	0 (0)	0.762
	1‐2 times	78 (44.1)	93 (52.5)				
	3‐5 times	13 (7.3)	12 (6.8)				
	6–10 times	3 (1.7)	1 (0.6)				
	More than 10 times	1 (0.6)	0				
When in the sun, how often do you use sunscreens?	<30 min	97 (54.8)	92 (52.0)	0 (1)	0 (1)	0 (0)	0.814
	30 min–1 h	50 (28.2)	54 (30.5)				
	1–2 h	19 (10.7)	27 (15.3)				
	2–3 h	8 (4.5)	3 (1.7)				
	>3 h	3 (1.7)	1 (0.6)				
How often do you sunbathe with the intention to get tanned?	Never	58 (32.8)	63 (35.6)	1 (2)	1 (2)	0 (0)	0.212
	Seldom	61 (34.5)	62 (35.0)				
	Occasionally	44 (24.9)	36 (20.3)				
	Often	10 (5.6)	14 (7.9)				
	Always	4 (2.3)	2 (1.1)				
How long do you usually stay in the sun between 11 AM and 3 PM on a typical day off?	<30 min	41 (23.2)	53 (29.9)	1 (1)	1 (2)	0 (1)	**0.007**
	30 min–1 h	61 (34.5)	69 (39.0)				
	1–2 h	49 (27.7)	35 (19.8)				
	2–3 h	17 (9.6)	12 (6.8)				
	>3 h	7 (4.0)	7 (4.0)				
How often do you take a holiday with the intention of spending more time in the sun?	Never	22 (12.4)	27 (15.3)	2 (1)	1 (1)	1 (0)	**0.008**
	Seldom	56 (31.6)	62 (35.0)				
	1–2 weeks a year	87 (49.2)	82 (46.3)				
	3–5 weeks a year	12 (6.8)	6 (3.4)				
	>5 weeks a year	0	0				
When in the sun, how often do you use covering clothing for sun protection?	Always	23 (13.0)	22 (12.4)	2 (2)	2 (2)	0 (1)	0.224
	Often	57 (32.2)	49 (27.7)				
	Occasionally	51 (28.8)	52 (29.9)				
	Seldom	31 (17.5)	37 (20.9)				
	Never	15 (8.5)	16 (9.0)				
How often do you stay indoors or in shade in order to protect yourself from the sun?	Always	11 (6.2)	15 (8.5)	2 (1)	2 (1)	0 (1)	**0.01**
	Often	67 (37.9)	71 (40.1)				
	Occasionally	59 (33.3)	65 (36.7)				
	Seldom	29 (16.4)	22 (12.4)				
	Never	11 (6.2)	4 (2.3)				
When in the sun, how often do you use a sun hat or cap for sun protection?	Always	26 (14.7)	29 (16.4)	2 (2)	2 (2)	0 (2)	0.774
	Often	46 (26.0)	39 (22.0)				
	Occasionally	38 (21.5)	41 (23.2)				
	Seldom	28 (15.8)	36 (20.3)				
	Never	39 (22.0)	32 (18.1)				
***Total SEPI part 1 score***				11 (7)	11 (6)	0 (4)	0.124

*Note*: SEPI Part 1 evaluates sun‐related habits; higher scores reflect higher risk behaviour. Each item is scored 0‐4, maximum score 32. *p* values less than 0.05 are highlighted in bold.

**TABLE 3:** Results from SEPI part 2 questionnaire items: Propensity to increase sun protection: Higher scores reflect lower readiness to improve behaviour.

Statement	Response categories	Frequency in survey 1, *n* (%)	Frequency in survey 2, *n* (%)	Survey 1 median score (IQR)	Survey 2 median score, (IQR)	Difference in scores, median (IQR)	*p* value
Sunscreens	I Have never thought of using sunscreens.	2 (1.1)	1 (0.6)	0 (1)	0 (1)	0 (0)	0.177
	I Could think of using sunscreens.	12 (6.8)	13 (7.3)				
	I Intend to start using sunscreens.	15 (8.5)	8 (4.5)				
	I Have started to use sunscreens.	31 (17.5)	30 (16.9				
	I Have for a long time used sunscreens.	117 (66.1)	125 (70.6)				
The shade	I Have never thought of trying to stay in the shade during the hours of strongest sun light.	10 (5.6)	3 (1.7)	1 (3)	1 (3)	0 (1)	**0.02**
	I Could think of trying to stay in the shade during the hours of strongest sun light.	40 (22.6)	41 (23.2)				
	I Intend to start trying to stay in the shade during the hours of strongest sun light.	28 (15.8)	21 (11.9)				
	I Have started trying to stay in the shade during the hours of strongest sun light.	38 (21.5)	41 (23.2)				
	I Have for a long time tried to stay in the shade during the hours of strongest sun light.	61 (34.5)	71 (40.1)				
Sunbathing	I Have never thought of giving up sunbathing	47 (26.6)	37 (20.9)	3 (4)	1 (3)	2 (1)	**0.003**
	I Could think of giving up sunbathing	42 (23.7)	39 (22.0)				
	I Intend to give up sunbathing.	3 (1.7)	3 (1.7)				
	I Have recently given up sunbathing.	10 (5.7)	11 (6.2)				
	I Have for a long time avoided sunbathing	75 (42.4)	87 (49.2)				
Covering clothes	I Have never thought of using covering clothes for sun protection.	19 (10.7)	16 (9.0)	2 (3)	1 (2)	0 (2)	0.219
	I Could think of using covering clothes for sun protection.	58 (32.8)	43 (24.3)				
	I Intend to start using covering clothes for sun protection.	16 (9.0)	27 (15.3)				
	I Have started to use covering clothes for sun protection.	34 (19.2)	49 (27.7)				
	I Have for a long time used covering clothes for sun protection.	50 (28.2)	42 (23.7)				
Sun hat or cap	I Have never thought of using a sun hat or cap for sun protection.	24 (13.6)	18 (10.2)	2 (3)	2 (2)	0 (1)	0.371
	I Could think of using a sun hat or cap for sun protection. I Intend to start using a sun hat or cap for sun protection.	51 (28.8)	46 (26.0)				
		31 (17.5)	37 (20.9)				
	I Have started to use a sun hat or cap for sun protection. I Have for a long time used a sun hat or cap for sun protection.	27 (15.3)	40 (22.6)				
		44 (24.9)	36 (20.3)				
***Total SEPI part 2 score***				7 (8)	6 (6)	1 (4)	**0.004**

*Note*: Each item has a maximum score of 4, for the response least open to changing behaviour; the maximum total score is 20. *p* values less than 0.05 are highlighted in bold.

### 3.2 Beliefs surrounding sun protection and tanning

Table 4 illustrates results from both surveys, for all participants, regarding further questions asked beyond the SEPI questionnaire items; 96% and 95% in Surveys 1 and 2 agreed that sun protective measures decreased lifetime risk of skin cancer development; 68% in Survey 1 agreed that tanned skin reflected damaged skin; this increased to 74% in the post‐campaign survey. Sunscreen preferences remained relatively static, with 78% and 81% in surveys 1 and 2 respectively reported preference for SPF30 or higher when using sunscreens. Similarly, over half of respondents in each survey (54% and 56% respectively) reported always wearing protective sunglasses when in the sun; 44% in Survey 1% and 41% in Survey 2 disagreed that the same sun protective behaviours should be used in Ireland compared to when in sunnier countries. When asked about sun bed use in survey 2, 40% of respondents reported previous sun bed use, all at least on more than 10 occasions. Only 1% (*n* = 8) reported current or active sunbed use; 96% acknowledged that sunbed use increased the risk of skin cancer.

**TABLE 4:** Survey questions of beliefs surrounding sun protection and skin cancer risk, wearing sunglasses, skin protection in Ireland versus abroad, and sunbed use.

Survey question/statement	Response options	Survey 1 (*n* = 854) *n* (percentage of responses)	Survey 2 (*n* = 704) *n* (percentage of responses)
Using sun protection, avoiding sunburn and reducing the amount of time I spend in the sun at any age can decrease my lifetime risk of skin cancer	Strongly agree	720 (84.31%)	587 (83.98%)
	Somewhat agree	105 (12.3%)	80 (11.44%)
	Neutral	22 (2.58%)	26 (3.72%)
	Somewhat disagree	4 (0.47%)	2 (0.29%)
	Strongly disagree	3 (0.35%)	4 (0.57%)
My lifetime sun exposure is linked to my skin cancer risk	Strongly agree	619 (72.74%)	546 (78%)
	Somewhat agree	161 (18.92%)	105 (15.14%)
	Neutral	46 (5.41%)	38 (5.43%)
	Somewhat disagree	14 (1.65%)	6 (0.86%)
	Strongly disagree	11 (1.29%)	4 (0.57%)
Tanned skin is damaged skin	Strongly agree	298 (35.02%)	291 (41.63%)
	Somewhat agree	282 (33.14%)	230 (32.9%)
	Neutral	184 (21.62%)	106 (15.16%)
	Somewhat disagree	63 (7.4%)	58 (8.3%)
	Strongly disagree	24 (2.82%)	14 (2%)
How often do you wear sunglasses when in the sun?	Always	459 (53.75%)	391 (56.26%)
	Often	237 (27.75%)	183 (26.33%)
	Occasionally	97 (11.36%)	70 (10.07%)
	Seldom	29 (3.40%)	30 (4.32%)
	Never	32 (3.75%)	21 (3.02%)
I Protect my skin from the sun in Ireland Apr‐Sep as I would abroad in hot sunny countries	Strongly agree	187 (21.95%)	191 (27.32%)
	Somewhat agree	284 (33.33%)	218 (31.19%)
	Neutral	145 (17.02%)	94 (13.45%)
	Somewhat disagree	170 (19.95%)	146 (20.89%)
	Strongly disagree	66 (7.75%)	50 (7.15%)
Have you used sunbeds?	No, I have never used a sunbed		409 (58.51%)
	Yes, I currently use sunbeds		8 (1.14%)
	Yes, I have used them in the past		282 (40.34%)
If you have used sunbeds how many times in your lifetime have you used a sunbed? (Counting each individual visit)	Greater than 10 times		150 (47.92%)
	10‐30 times		120 (38.34%)
	30‐100 times		37 (11.82%)
	Greater than 100 times		6 (1.92%)
Sunbed use increases the risk of skin cancer	True		668 (95.7%)
	False		3 (0.43%)
	Don't know		27 (3.87%)
Sun exposure (both natural and from sunbeds) increases skin ageing	True		672 (96.28%)
	False		3 (0.43%)
	Don't know		23 (3.3%)
If you use sunscreen what sun protection factor do you apply?	Less than 15	18 (2.12%)	7 (1.01%)
	15	71 (8.35%)	53 (7.64%)
	20	97 (11.41%)	70 (10.09%)
	30	356 (41.88%)	285 (41.07%)
	50	308 (36.24%)	279 (40.20%)
How would you describe your confidence in correctly protecting your skin from the sun?	Very confident		176 (25.14%)
	Somewhat confident		411 (58.71%)
	Neutral		91 (13%)
	Somewhat not confident		21 (3%)
	Not confident at all		1 (0.14%)
How would you describe your confidence in communicating sun protection messages for example, ‘SunSmart’ advice to friends and family?	Very confident		213 (30.69%)
	Somewhat confident		320 (46.11%)
	Neutral		123 (17.72%)
	Somewhat not confident		28 (4.03%)
	Not confident at all		10 (1.44%)
Have you heard of the healthy Ireland SunSmart code? (Slip, Slop, Slap, seek, Slide)	Yes	280 (32.98%)	381 (54.98%)
	No	526 (61.96%)	275 (39.68%)
	I don't remember	43 (5.06%)	37 (5.34%)
Have you seen this year's ULHG SunSmart campaign across our 6 hospital sites?	Yes		563 (80.66%)
	No		135 (19.34%)
What most influences you to follow sun protective measures?	I Worry about the risk of skin cancer		297 (42.49%)
	I don't want to get sunburnt		254 (36.34%)
	I Worry about skin ageing		88 (12.59%)

Motivation to follow sun protection advice was primarily driven by fear of skin cancer development (42%), followed by avoiding sunburn (36%) and concern regarding premature skin ageing (13%). On questioning about perceived barriers to sun protection participants most frequently reported forgetting to protect skin (33%), dislike of covering clothing in warm weather (33%), preference to tan (25%), dislike of sunscreen sensation (19%) and inconvenience (15%).

Pre‐campaign only 33% reported awareness of SunSmart messaging. This increased to 55% post‐campaign; 80% of participants in survey 2 confirmed they had seen the campaign; 259 of these (46%) saw videos displayed across screens on hospital sites, 25% on social media and 25.5% on the hospital staff mobile app.

### 3.3 Confidence to communicate sun protection messaging

A slight improvement in reported confidence levels in sun protection was observed in overall survey results. In Survey 1 (*n* = 857), 59% (*n* = 503) described themselves as ‘somewhat confident’ and 21% (*n* = 183) described themselves ‘very confident’ in protecting their skin from the sun. These figures increased in Survey 2 (*n* = 704) to 58% (*n* = 411) and 25% (*n* = 176) respectively; 64% reported the campaign improved how they protect their skin; 79% reported raised awareness of skin cancer, and 65% said it influenced them to discuss sun protection with others.

## 4 DISCUSSION

Best sun protective habits pre‐campaign included use of sunscreens and avoiding sun burn, while the wearing of hats, covering clothing and seeking shade were least adopted with higher total SEPI scores. Similar preferences have been described in previous studies, in which sunscreen use has been the first preference in sun protection, due to ease of use and effective prevention of sun burn.^17^ While it is typically the most emphasised method of sun protection, covering clothing and hats may be more effective.^18^

In the second survey, following the summer campaign, there were small changes in median scores for different domains of sun protection habits and readiness to improve safe sun behaviours. Analysis of scores for matched participants showed non‐statistically significant improvement in most individual sun‐protective behaviours, but significantly reduced time spent in the midday sun (*p* = 0.007), reduced time spent on sun‐seeking holidays (*p* = 0.008), and improved readiness to reduce sunbathing (*p* = 0.003) and seek shade (*p* = 0.02). Only minor improvement in hat or covering clothing use, and willingness to adopt these, was observed in this study. The campaign highlighted not only the risks of sun exposure in relation to skin cancer development, but also accelerated skin ageing, and specifically cautioned against sunbathing. Whether this difference can be attributed to the campaign is difficult to determine, though most responders in the second survey had remarked that they had not seen other campaigns. The clinical relevance of small improvements in use of sun protective measures and willingness to adopt further changes in these is difficult to determine, as ultimately longer term larger studies comparing incidence of skin cancer after wider rollout of campaigns will be necessary to determine if there is clinical effect in this primary prevention measure. This has been demonstrated in relation to the Australian SunSmart campaign with associated reduced incidence of melanoma in younger age groups in the decades following introduction of the initiative.^23^

Interestingly, almost all respondents acknowledged that sun exposure and sunbed use increased the risk of skin cancer development and premature skin ageing, 26% did not associate skin tanning with skin damage, and 40% reported previous sunbed use. Post‐campaign, despite the advice for adequate sun protection especially between the months of April to September (when UV index is typically 3 or greater), over 40% considered the requirement for sun protection to be less in Ireland than when in other countries.

Design, delivery and promotion of public health messaging and primary prevention of disease must be accessible across all of society, and ULHG staff represent a very unique broad cohort to survey within the general population. The positive impact of health messaging on healthcare workers' personal beliefs and attitudes towards sun protection, and their confidence in discussing safe sun behaviours with their patients, families and friends as found in the post‐campaign survey results represents a potential for broader impact.

The medical literature surrounding the attitudes of healthcare workers towards sun safety is relatively limited. A Spanish study,^10^ conducted on staff across multiple professions, found that nurses tended to frequent the beach for sunbathing more so than doctors; and those in primary healthcare had superior sun protective behaviours than those in secondary care. A survey of US primary care clinicians found that many regularly counselled patients on sun protection, and identified significant barriers to doing so, however the attitudes of the staff themselves was not assessed.^19^ Though this study did not stratify findings for different professions, increasing sun protection awareness and encouraging adoption of safe sun practices in all health staff, even those not directly patient facing, has potential for further benefit to society; these individuals are ideally positioned to communicate such messaging with friends, relatives and any patients they encounter.^20^, ^21^

Historically in the UK and Ireland, health promotion campaigns surrounding photoprotection and skin cancer found that while there was high recognition of the association of sun exposure and development of skin cancer, adoption of sun protective measures was limited most commonly to application of sunscreens.^22^ The SunSmart campaign, an internationally recognised skin cancer prevention health promotion programme was first introduced in 1988 in Australia following the original ‘Slip! Slop! Slap!’ Campaign in the early 1980s.^23^ The campaign has been run there for over 30 years, where it is widely adopted.^24^ A large population survey^25^ showed increased sun protective behaviour in response to the campaign, particularly shortly after the SunSmart programme was initiated, and estimated that the campaign contributed to the reduction of melanoma incidence among younger cohorts. In the UK, the SunSmart campaign, adapted from the original Australian model, has been the national skin cancer prevention campaign since 2003.^5^ In the year of its introduction, Cancer Research UK conducted a survey which highlighted the need for increased knowledge of sun‐safe attitudes and behaviour and attitudes towards sun‐tanning.^26^ In the Republic of Ireland, where this study took place, the National Skin Cancer Prevention Plan which incorporates SunSmart messaging was first introduced in 2019.^9^ A recent critical evaluation of the campaign in the UK has determined however that, unlike Australia, it has not been effective there in improving healthy sun protection habits, and found that it was not relevant to Black and minority ethnic groups.^27^ Possible explanations for the suboptimal impact mentioned included lack of a comprehensive approach in public health messaging, and lack of funding, social and environmental influences.

Limitations of this study include the relatively short duration of the campaign (3 months), which may represent the challenge to change attitudes and beliefs surrounding sun exposure and protection. Interventions targeting sun protection attitudes can vary in duration^28^; those using the SEPI questionnaire for shorter term studies are limited, with one randomised controlled trial in Brazil adopting it as the basis for their survey evaluating the effect of a face‐ageing mobile application on behaviour of Brazilian school students.^29^ While invitation to participate in surveys was universally distributed across all hospital‐wide platforms, participation of males and medical staff was proportionally low. Males have been recognised as less likely than females to engage with sun protection and skin cancer awareness campaigns, and females may be more receptive to messaging about behaviours to improve health,^30^ which may in part be an explanation for this. Low numbers of doctors participated in the study (4.3% in Survey 1% and 4.6% in Survey 2), which is lower than in other studies regarding sun protection among healthcare staff.^11^, ^31^ Actual figures for staff demographics were not available from hospital management for comparison to determine if this proportion of study participants was representative of the overall staff population. This skew in representation of mostly female participants, and low numbers of doctors, is nonetheless a further limitation as findings may not accurately be extrapolated to this entire working population. Other limitations include the method of participant recruitment, which was directly impacted by the cyber‐attack on the national healthcare system; challenges in identifying participants who completed both surveys (matched by an external statistician based on email addresses entered in survey software), and bias in that those choosing to participate may have had a greater background interest in sun protection or safer sun‐protective behaviours at baseline.

Overall, the digital campaign was well‐received, and the post‐campaign survey demonstrated changes in attitudes and behaviour surrounding sun protection. These findings lend support for the potential to leverage novel digital health promotion initiatives into the future. Statistically significant improvements were seen for reducing sunbathing behaviours and seeking shade. The campaign and surveys have been planned to be repeated after a 2‐year period to reassess the longer‐term changes in these same attitudes and behaviours in this population. Further emphasis of the multiple aspects of sun protection, including covering clothing and the wearing of hats, should be made in addition to skin cancer risk awareness in future sun safety messaging.

## CONFLICT OF INTEREST STATEMENT

The authors declare no conflicts of interest.

## AUTHOR CONTRIBUTION

**Emma Porter**: Conceptualization; Data curation; Formal analysis; Investigation; Methodology; Project administration; Visualization; Writing – original draft; Writing – review & editing. **Siobhan Rafferty**: Writing – review & editing. **Michelle Dolan**: Conceptualization; Resources; Writing – review & editing. **David McMahon**: Conceptualization; Resources. **Ali Sheikhi**: Formal analysis; Validation. **Sinead Field**: Conceptualization; Project administration; Supervision; Writing – review & editing. **Evelyn Power**: Conceptualization; Funding acquisition; Investigation; Project administration; Resources; Supervision; Visualization; Writing – review & editing.

## ETHICS STATEMENT

Ethical approval was granted by the University of Limerick Hospitals Group (ULHG) research ethics committee.

## Supporting information

Supplementary Material

Supplementary Material

## Data Availability

The data that support the findings of this study are available from the corresponding author upon reasonable request.
