# Supplementary material for: The impact of a novel digital sun protection campaign on sun‐related attitudes and behaviours of healthcare workers: A prospective observational study
Source: Skin Health Dis. 2023 Jun 8;4(6):e256. doi: 10.1002/ski2.256 (PMC11608873; doi:10.1002/ski2.256)
Supplement: Supplementary file 2 — Supplementary Material [file SKI2-4-e256-s001.docx]

**About You**

1. **Age**

- 18-24
- 25-34
- 35-44
- 45-54
- 55-64
- 65-74
- 75+

1. **Gender**

- Male
- Female
- Non-binary
- Prefer not to say

1. **Marital Status**

- Single
- Married/civil partnership
- Separated
- Divorced
- Widowed
- Prefer not to say

1. **What is the highest level of education you have completed to date?**

- No formal education
- Primary school
- Lower secondary school (eg Junior Certificate)
- Upper secondary school (eg Leaving Certificate)
- Technical or vocational qualification (eg apprenticeship)
- Postgraduate certificate or diploma
- Third level/University (Bachelor) degree
- Masters degree
- Doctorate (PhD)
- Prefer not to say

1. **Your role in the hospital**

- Medical
- Nursing
- Allied health and related fields e.g.
  - - Care assistant/attendant
    - Laboratory
    - Occupational therapist
    - Paramedic/ambulance
    - Pharmacist
    - Physiotherapist
    - Social worker
- Hospital management
- Hospital administration
- Health and safety
- Catering
- Grounds personnel
- Maintenance
- Porter
- Stores personnel
- Religious affairs/chaplaincy
- Other (specify) _____________

1. **Where do you mostly work?**

- Indoors
- Outdoors
- Other

1. **If you work outdoors, how many hours per week do you spend outdoors?**

- ______________

1. **Which of the following statements best describes what happens to your skin if you were to be exposed to the sun without sun protection?**

- Always burns, never tans
- Usually burns, tans minimally
- Burns moderately, tans uniformly
- Burns minimally, always tans (olive-moderate brown skin)
- Rarely burns, tans very easily (brown-dark brown skin)
- Never burns, always tans (dark brown to black skin)

**Your Skin and Sun Protection – Part 1**

The following questions were asked in the first questionnaire sent in April; we would be grateful if you could answer them again today.

1. **How often do you sunbathe with the intention to get tanned?**

- Never
- Seldom
- Occasionally
- Often
- Always

1. **How many times have you been sunburnt (redness and pain) during the last 12 months?**

- None
- 1-2 times
- 3-5 times
- 6-10 times
- More than 10 times

1. **How long do you usually stay in the sun (on average), between 11 am and 3 pm, on a typical day-off?**

- < 30 min
- 30 min – 1 hour
- 1-2 hours
- 2-3 hours
- > 3 hours

1. **How often do you take a holiday with the intention of spending more time in the sun?**

- Never
- Seldom
- 1-2 weeks a year
- 3-5 weeks a year
- > 5 weeks a year

1. **When in the sun, how often do you use sunscreens?**

- Always
- Often
- Occasionally
- Seldom
- Never

1. **When in the sun, how often do you use a sun hat or cap for sun protection?**

- Always
- Often
- Occasionally
- Seldom
- Never

1. **When in the sun, how often do you use covering clothing for sun protection?**

- Always
- Often
- Occasionally
- Seldom
- Never

1. **How often do you stay indoors or in the shade in order to protect yourself from the sun?**

- Always
- Often
- Occasionally
- Seldom
- Never

**Choose for each item which statement is most consistent with your own thoughts/ intentions:**

1. **Sunbathing:**

- *I have never thought of* **giving up sunbathing.**
- *I could think of* **giving up sunbathing.**
- *I intend to* **give up sunbathing.**
- *I have recently* **given up sunbathing.**
- *I have for a long time* **avoided sunbathing**

1. **Sunscreens:**

- *I have never thought of* **using sunscreens.**
- *I could think of* **using sunscreens.**
- *I intend to start* **using sunscreens.**
- *I have started to* **use sunscreens.**
- *I have for a long time* **used sunscreens.**

1. **Covering clothes:**

- *I have never thought of* **using covering clothes for sun protection**.
- *I could think of* **using** **covering clothes for sun protection.**
- *I intend to start* **using** **covering clothes for sun protection.**
- *I have started to* **use covering clothes for sun protection.**
- *I have for a long time* **used covering clothes for sun protection.**

1. **Sun hat or cap:**

- *I have never thought of* **using a sun hat or cap for sun protection.**
- *I could think of* **using a sun hat or cap for sun protection**.
- *I intend to start* **using a sun hat or cap for sun protection.**
- *I have started to* **use a sun hat or cap for sun protection.**
- *I have for a long time* **used a sun hat or cap for sun protection.**

1. **The shade:**

- *I have never thought of* **trying to stay in the shade during the hours of strongest sun light.**
- *I could think of* **trying to stay in the shade during the hours of strongest sun light.**
- *I intend to start* **trying to stay in the shade during the hours of strongest sun light.**
- *I have started* **trying to stay in the shade during the hours of strongest sun light.**
- *I have for a long time* **tried to stay in the shade during the hours of strongest sun light.**

**Your Skin and Sun Protection – Part 2**

1. **If you use sunscreen, what sun protection factor (SPF) do you apply?**

- Less than 15
- 15
- 20
- 30
- 50

1. **Have you used sunbeds?**

- No, I have never used a sunbed
- Yes, I currently use sunbeds
- Yes, I have used them in the past

1. **If you have used sunbeds, how many times in your lifetime have you used a sunbed? (counting each individual visit)**

- Greater than10 times
- 10-30 times
- 30-100 times
- Greater than 100 times

1. **How often do you wear sunglasses when in the sun?**

- Always
- Often
- Occasionally
- Seldom
- Never

1. **My lifetime sun exposure is linked to my skin cancer risk**

- Strongly Agree
- Somewhat Agree
- Neutral
- Somewhat Disagree
- Strongly Disagree

1. **Using sun protection, avoiding sunburn and reducing the amount of time I spend in the sun at any age can decrease my lifetime risk of skin cancer**

- Strongly Agree
- Somewhat Agree
- Neutral
- Somewhat Disagree
- Strongly Disagree

1. **Tanned skin is damaged skin**

- Strongly Agree
- Somewhat Agree
- Neutral
- Somewhat Disagree
- Strongly Disagree

1. **I protect my skin from the sun in Ireland April-September as I would abroad in hot sunny countries**

- Strongly Agree
- Somewhat Agree
- Neutral
- Somewhat Disagree
- Strongly Disagree

1. **Children’s skin is more sensitive to UV damage than adults**

- True
- False
- Don’t know

1. **Sun exposure (both natural and from sunbeds) increases skin aging**

- True
- False
- Don’t know

1. **Sunbed use increases the risk of skin cancer**

- True
- False
- Don’t know

1. **What most influences you to follow sun protective measures?**

- I don’t want to get sunburnt
- I worry about the risk of skin cancer
- I don’t like a sun tan / darkening of my skin
- I worry about my skin aging
- Advice from family/friends
- Advice from a health professional
- Health promotion campaigns
- Other (specify) _________

1. **How would you describe your confidence in correctly protecting your skin from the sun?**

- Very confident
- Somewhat confident
- Neutral
- Somewhat not confident
- Not confident at all

1. **What barriers do you think exist that prevent you from protecting your skin from the sun? (tick all that apply – multiple can be selected)**
   - I don’t think there are barriers
   - It is inconvenient
   - I like being tanned
   - Cost of sunscreens
   - I don’t like the feeling of sunscreens on my skin
   - Wearing covering clothes is too hot in the summer
   - Fashion reasons (eg wearing a wide-brimmed hat)
   - Peer pressure
   - It’s not worth the trouble
   - I forget
   - Lack of shade outdoors
   - I worry I won’t get adequate Vitamin D from the sun
   - Other (specify) _________
2. **How would you describe your confidence in communicating sun protection messages e.g. ‘SunSmart’ advice to friends and family?**

- Very confident
- Somewhat confident
- Neutral
- Somewhat not confident
- Not confident at all

1. **If you work with patients, how would you describe your confidence in communicating sun protection messages e.g. ‘SunSmart’ advice to them?**

- Very confident
- Somewhat confident
- Neutral
- Somewhat not confident
- Not confident at all
- I do not work with patients

1. **Have you heard of the Healthy Ireland SunSmart code? (Slip, Slop, Slap, Seek, Slide)**

- Yes
- No
- I don’t remember

1. **If you have seen or heard campaigns about sun protection in the last year, where did you see/hear the campaign? (select all that apply)**

| I have not seen/heard any | ❑ | Other (please specify) | ❑ |
| --- | --- | --- | --- |
| Television | ❑ | Magazines | ❑ |
| Newspaper | ❑ | Workplace | ❑ |
| Radio | ❑ | Health professionals (GP/nurse etc) | ❑ |
| Facebook | ❑ | Pharmacy | ❑ |
| Instagram | ❑ | School | ❑ |
| Twitter | ❑ | Family/friends/word of mouth | ❑ |
| Other social media platform | ❑ | Posters/leaflets in public places | ❑ |

1. **Have you seen this year’s ULHG SunSmart Campaign across our 6 hospital sites?**

- Yes
- No

1. **If yes, where have you seen the campaign? (select all that apply)**

- Visual display units on hospital campus (ULHG)
- On ULHG’s social media
- ULHG Staff app / NCHD app
- Other social media
- Posters in staff areas of ULHG
- Local newspaper
- Local radio
- National newspaper
- National radio
- Not applicable / I have not seen the campaign
- Other (please specify) ______

1. **Do you consider that the ULHG SunSmart campaign has improved how you protect your skin from the sun?**

- Yes
- No
- Not sure

1. **Do you consider that the campaign has raised your awareness of skin cancer?**

- Yes
- No
- Not sure

1. **Do you think that the campaign has influenced you to discuss sun awareness and sun protection with others?**

- Yes
- No
- Not sure

1. **Any further comments or suggestions for delivery of sun protection campaigns?**

|  |
| --- |

**________________________________________________________**

This year **‘ULHG’s SunSmart Campaign** has been running on the visual display units and social media in ULHG in conjunction with the Irish Skin Foundation and the NCCP.

Thank you for your completing this survey. If you would like to be entered into a draw for a raffle prizes, please include your email address below, otherwise click ‘Finish’.

**Note: If you completed Part I of this survey, please use the same email address for the prize draw.**

| **EMAIL ADDRESS:** |
| --- |
